# Effectiveness of continuous glucose monitoring on maternal and neonatal outcomes in gestational diabetes mellitus: a systematic review and meta-analysis

**DOI:** 10.1186/s12884-026-08663-8

**Published:** 2026-01-30

**Authors:** Renuka Suvarna, Sahana Shetty

**Affiliations:** Department of Endocrinology, Kasturba Medical College, Manipal Academy of Higher Education, Manipal, Karnataka 576104 India

**Keywords:** CGM, GDM, SMBG, Maternal outcomes, Neonatal outcomes

## Abstract

**Background:**

Continuous Glucose Monitoring (CGM) by providing real-time glucose data and glucose trends is a useful tool in the management of gestational diabetes mellitus (GDM). This systematic review and meta-analysis of Randomized Clinical Trials (RCTs) evaluated the impact of CGM on maternal and neonatal outcomes in women with GDM.

**Methods:**

A comprehensive search of PubMed, Cochrane Library, EMBASE, and Scopus identified RCTs comparing CGM with SMBG in women with GDM. Outcomes were analyzed using mean differences and odds ratios, with meta-analysis performed in RevMan 5.4.

**Results:**

Five studies were included in this review. Maternal outcomes showed a significant reduction in HbA1c [MD: -0.21% (-0.39, -0.03) *p* = 0.02] with CGM compared to Self-Monitoring of Blood Glucose (SMBG). Neonatal outcomes indicated a lower rate of macrosomia [OR: 0.50 (0.40, 0.61) *p* < 0.00001] and reduced birth weight [MD: -151.68 gm (-237.21, -66.14) *p* = 0.0005] in the CGM group. However, no significant differences were observed in other secondary outcomes.

**Conclusion:**

The study demonstrated that the CGM group had a significantly lower HbA1c, with lower rates of macrosomia and reduced birth weights compared to SMBG. While CGM is effective for improving outcomes in GDM, further research is needed to establish pregnancy-specific glucose targets and address cost and accessibility challenges.

**Supplementary Information:**

The online version contains supplementary material available at 10.1186/s12884-026-08663-8.

## Introduction

Gestational diabetes mellitus (GDM) is a metabolic disorder characterized by elevated blood glucose levels first identified during pregnancy. GDM can lead to serious maternal and neonatal complications if not well managed. It increases the risk of preeclampsia, cesarean delivery, and future type 2 diabetes and cardiovascular disease in mothers. For infants, GDM raises the likelihood of macrosomia, birth injuries, neonatal hypoglycemia, and respiratory distress [1]. GDM is a significant public health concern worldwide. According to the International Diabetes Federation (IDF) Diabetes Atlas (2025), approximately 1 out of 6 live births are affected specifically by GDM. Globally, about 16.7% of births in women aged 20–49 is affected by hyperglycemia during pregnancy, with GDM contributing to the majority of these cases [2]. The prevalence varies widely depending on diagnostic criteria, geographic regions, and population characteristics [3]. It ranges from 10.1% to 28%, with higher rates reported in Southeast Asia and the Middle East due to genetic predisposition, urbanization, and lifestyle changes [4].

Continuous glucose monitoring (CGM) is a monitoring tool in the management of GDM, offering real-time insights into glycemic patterns and enabling improved glucose control compared to traditional Self-Monitoring of Blood Glucose (SMBG) [5]. In pregnant women with GDM, these insights are critical for maintaining tight glycemic control and reducing maternal and fetal complications [6]. CGM has emerged as a valuable tool for optimizing glycemic management in GDM. By providing real-time glucose data, CGM supports personalized treatment plans, reducing the risk of complications, and improving maternal and neonatal outcomes. However, challenges like affordability and accessibility need to be addressed to ensure widespread adoption [7].

The utility of CGM in GDM and its impact on maternal and neonatal outcomes is a critical area of research with direct implications for clinical practice and healthcare policy. Although CGM is increasingly recognized for its ability to optimize glycemic control, its clinical utility in GDM remains debated. The current controversies include inconsistent evidence on maternal and neonatal outcomes, the absence of pregnancy-specific glycemic targets, the lack of consensus on timing and frequency of use, and the concerns about sensor accuracy, the cost, and generalizability of data [8]. These uncertainties have led to cautious recommendations in guidelines, with SMBG still considered the standard of care. While several trials and observational studies have reported improvements in HbA1c, reductions in macrosomia, and favorable neonatal outcomes, others have found little or no advantage compared with traditional SMBG. These discrepancies raise concerns regarding the consistency of CGM’s benefits, its economic feasibility, and its relevance to pregnancy. Moreover, much of the existing evidence is limited by heterogeneous study populations that combine women with type 1, type 2, and gestational diabetes, thereby diluting the conclusions for GDM. Against this backdrop, the present meta-analysis provides a targeted evaluation of CGM in GDM, aiming to synthesize available data, resolve ongoing controversies, and generate clinically meaningful insights that can refine treatment strategies and inform public health policies tailored to gestational diabetes care.

## Methods

This metanalysis was carried out as per the PRISMA guidelines [9]. This systematic review has been registered with PROSPERO (CRD42025642304).

### Study selection

Randomized clinical trials (RCTs) comparing the effects of CGM with SMBG or standard care and focused on women diagnosed with GDM were the eligibility criteria for this study. Studies reporting maternal and neonatal outcomes were included. Non-randomized trials or studies lacking SMBG as comparators were excluded. Two reviewers independently screened titles, abstracts, and full-text articles according to predefined eligibility criteria. Any disagreements were resolved through discussion and consensus between the two reviewers.

Several bibliographic databases, including PubMed, EMBASE, the Cochrane Library, and Scopus, were searched electronically for RCTs. To find other pertinent studies, the reference lists of the included papers and additional systematic reviews were also examined. The literature search included studies published from January 2000 till December 2024. The search was limited to studies published in the English language. The search strategy encompassed combinations of the terms “gestational diabetes mellitus” OR “GDM” AND “continuous glucose monitoring” OR “CGM”. A detailed description of the search strategy is presented in Table S1.

### Outcome measures

The primary outcome measures are levels of HbA1c, macrosomia (defined as birth weight of 4000 g or more) and birth weight (low birth weight defined as birth weight less than 2500 g). The secondary outcome measures are gestational hypertension, the number of patients who require Insulin therapy, and caesarean sections. Neonatal outcomes include large for gestational age (LGA: birth weight > 90th percentile), small for gestational age (SGA: birth weight < 10th percentile), gestational age (GA) at birth, preterm delivery, neonatal hypoglycaemia, neonatal hyperbilirubinemia, Apgar score at 5 min, admission to the NICU and Respiratory distress syndrome (RDS).

### Data extraction

Two reviewers independently extracted the data using a standardized form. Any disagreements were resolved through discussion and consensus between the two reviewers. The following information was extracted: mean age, BMI, study design, sample size, country, author name, publication year, baseline HbA1c and fasting glucose readings, and sample size.

### Statistical analysis

Revman 5.4 was used to perform statistical analysis. Random-effects models were applied in the meta-analysis, and an I^2^ value greater than 50% was considered indicative of heterogeneity. Each outcome was represented by a forest plot, and pooled effects were computed using mean differences (MD) for continuous data and odds ratios (OR) for categorical data, along with 95% CIs. To assess publication bias, funnel plots were used. Sensitivity analysis and Egger’s test were performed for outcomes with three or more included studies.

### Risk of bias assessment

The Cochrane Risk of Bias Tools were employed to objectively assess the quality of included RCTs. Three categories were used to evaluate the potential sources of bias due to inappropriate experimental procedures or limitations in the sample during the study process: high risk, low risk, and unclear risk. The summary of the risk of bias was generated using Revman 5.4 software. One researcher performed the quality evaluations and any disagreements were discussed and resolved with the second researcher.

## Results

**Fig. 1:**
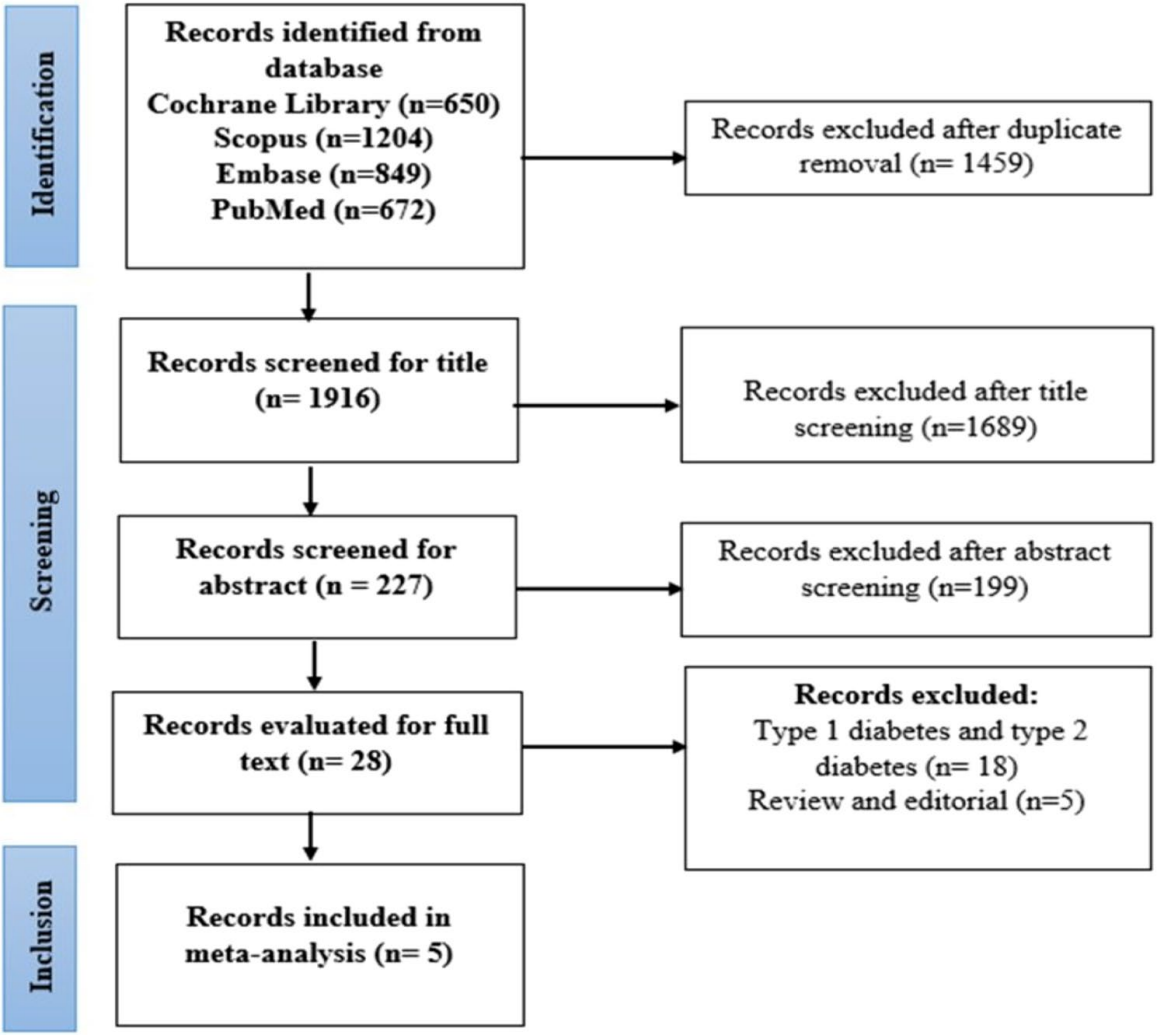
PRISMA flow chart of study selection

The database search yielded 3,375 papers in total, 1,916 of which were screened for titles and abstracts. Subsequently, 28 articles were selected for full-text review (Fig. 1). Two articles were excluded because one study included Type 1 and Type 2 diabetes alongside GDM, and the other compared blinded and non-blinded CGM. Ultimately, five articles were deemed eligible for meta-analysis.

### Study characteristics

The detailed study characteristics are explained in Table 1. In the study by Alfadhli E et al., GDM diagnosis followed the International Association of Diabetes in Pregnancy Study Groups (IADPSG) recommendations, with a 75-g OGTT conducted between 24 and 28 weeks of gestation [10]. Similarly, studies by Kestilä KK et al. [11], Lai M et al. [12], and Paramasivam SS et al. [13] diagnosed GDM based on one or more abnormal plasma glucose values during the 75-g OGTT performed at 24–28 weeks. In one study [11], the women underwent OGTT between 22 and 34 weeks if classified as high-risk per Finland’s evaluation system, including factors like BMI > 25 kg/m², age > 40 years, or previous gestational complications [11]. In one study [14], GDM was diagnosed using the American Diabetes Association (ADA) criteria based on a 75-g OGTT at 24–28 weeks. Throughout the study period, patients in the CGM group in every trial also had SMBG. The summary of diagnostic definitions and criteria of the included studies are presented in Table S2.

**Table 1:** Study characteristics of included articles

Author/year		Alfadhli E/2016	Kestila KK/2007	Lai M/2023	Paramasivam SS/2018	Wei Q/2016
Country		Saudi Arabia	Finland	China	Malaysia	China
CGMS device used		Guardian^®^ RT-CGM - Medtronic MiniMed	Medtronic MiniMed, (Northridge, CA, USA)	Medtronic Inc., (Northridge, CA)	Medtronic iPro2 Enlite	Gold Medtronic MiniMed (Northridge, CA, USA)
Diagnostic criteria		IADPSG	Criteria applied to a high-risk group according to the evaluation system used in Finland	IADPSG	Local guidelines	ADA
Sample size (n)	CGM	60	36	62	25	51
	SMBG	62	37	62	25	55
Age (years)	CGM	32.93 ± 5.70	32.6 ± 4.7	31.81 ± 4.33	32.8 ± 4.5	30.29 ± 3.60
	SMBG	34.15 ± 5.04	32.2 ± 5.7	31.77 ± 3.77	32.6 ± 4.9	29.96 ± 3.43
BMI (kg/m^2^)	CGM	31.13 ± 7.53	27.2 ± 3.9	22.23 ± 3.57	28.3 ± 4.8	NR
	SMBG	32.11 ± 5.74	26.1 ± 3.3	23.05 ± 3.53	27.3 ± 5.6	NR
GA during recruitment (weeks)	CGM	21.28 ± 6.26	28.7 ± 2.5	24–28	18.8 ± 3.7	24–28
	SMBG	23.95 ± 7.14	28.7 ± 2.3	24–28	20.7 ± 2.7	24–28
HbA1c (%)	CGM	5.6 ± 0.7	5.4 ± 0.4	4.94 ± 0.34	5.1 ± 0.3	5.7 ± 0.34
	SMBG	5.9 ± 0.8	5.3 ± 0.3	5.01 ± 0.36	5.3 ± 0.5	5.8 ± 0.29
FBS (mmol/L)	CGM	5.31 ± 1.06	5.5 ± 0.7	4.92 ± 0.56	4.4 ± 0.7	5.69 ± 0.58
	SMBG	5.01 ± 0.63	5.7 ± 1.0	4.97 ± 0.52	4.3 ± 0.5	5.67 ± 0.29

*CA* California, *NR* Not reported, *BMI* Body mass index, *FBS* Fasting blood sugar

In the study by Alfadhli et al. [10], women with GDM were divided into two groups: both performed SMBG four times daily for five days, while the intervention group also used RT-CGM during the same period. SMBG readings were used for CGM calibration and comparison. In the study by Kestilä et al. [11], CGM group used the CGMS for three consecutive days alongside routine SMBG. SMBG was performed at least four times daily—fasting and postprandially—and used for CGM calibration. Lai et al. [12] employed a blinded CGM system for 14 days at baseline, 4 weeks, and 8 weeks, while both groups performed SMBG four times daily for 3 consecutive days during each time point. In the trial by Paramasivam et al. [13], both groups performed SMBG at least four times daily—fasting and postprandial—throughout the study. The CGM group used a retrospective CGM system for 6–7 days at three time points: baseline, 30–32 weeks, and 36–38 weeks of gestation. SMBG readings were used for CGM calibration. Wei et al. [14] instructed participants to monitor glucose via SMBG four times daily (fasting and 1-hour post-meal) from diagnosis to delivery, while the CGM group wore sensors for 48–72 h and recorded additional readings at bedtime and 1 h before meals. All participants received dietary counseling in two studies [11, 13].

In all the included studies, treatment decisions for hyperglycemia in GDM women were primarily guided by SMBG values. In the study by Alfadhli et al. [10], although the intervention group utilized CGM as an educational tool, treatment adjustments were made based on SMBG data. Similarly, in Kestilä et al. [11], SMBG readings, measured at least four times daily, served as the basis for initiating or modifying insulin therapy, while CGM data was used retrospectively for evaluation. In Lai et al. [12], therapeutic decisions were also based on SMBG values, whereas CGM was used for outcome assessment. In Paramasivam et al. [13], all treatment decisions including insulin initiation and titration were made based on routine SMBG. Lastly, in the study by Wei et al. [14], glycemic management throughout pregnancy relied on SMBG readings. Across these trials, CGM mainly served an observational or supplementary role, while SMBG remained the primary tool for real-time clinical decision-making.

### Meta-analysis

**Fig. 2:**
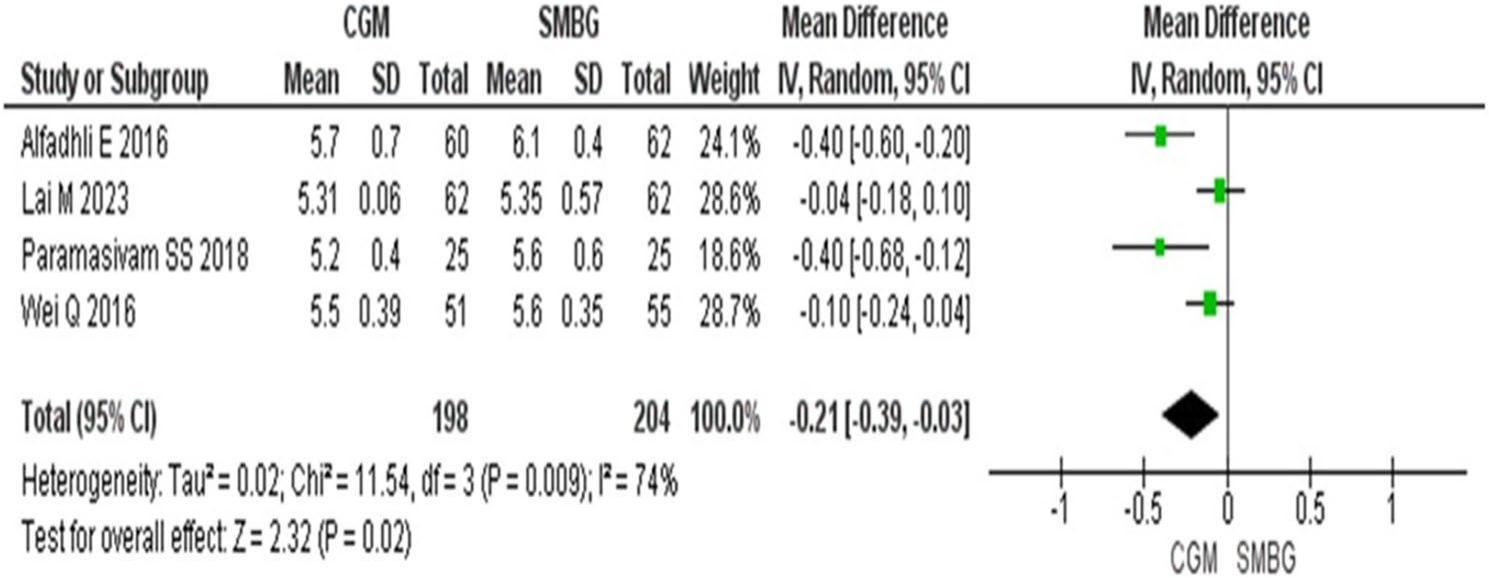
Forest plot for changes in the levels of HbA1c (%) in CGM vs. SMBG group

**Fig. 3:**
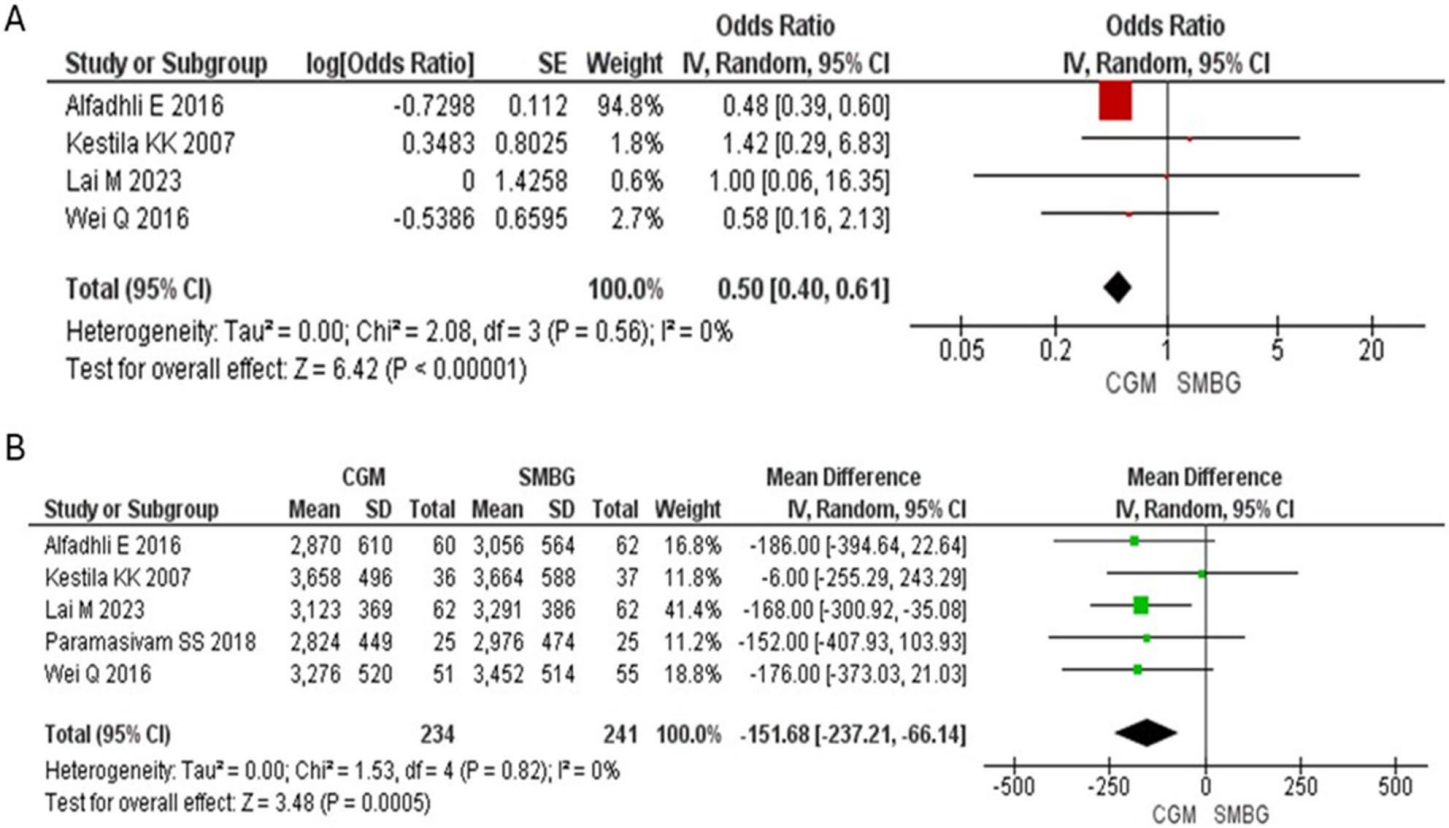
Forest plot for **A**) macrosomia and **B**) changes in the birth weight (gm) in CGM vs. SMBG group

A total of 475 participants from 5 included studies were analyzed, with 234 in the CGM group and 241 in the SMBG group for the meta-analysis. The meta-analysis of the four studies reporting levels of HbA1c showed significantly lower HbA1c in CGM group [MD: −0.21% (−0.39, −0.03) *p* = 0.02] compared to SMBG group (Fig. 2). Four studies reporting macrosomia [OR: 0.50 (0.40, 0.61) *p* < 0.00001] (Fig. 3A) and 5 studies reporting birth weight [MD: −151.68 gm (−237.21, −66.14) *p* = 0.0005] found significantly lower values in the CGM group compared to SMBG group (Fig. 3B). Macrosomia in one study was not included in the forest plot because macrosomia did not occur in this study.

The meta-analysis did not show any significant changes in secondary outcomes such as the gestational hypertension [OR: 2.41 (0.71, 8.13) *p* = 0.16], number of patients who required Insulin therapy [OR: 1.85 (0.98, 3.47) *p* = 0.06] and caesarean sections [OR: 0.88 (0.60, 1.28) *p* = 0.50] (Figure S1). Neonatal outcomes such as LGA [OR: 0.56 (0.29, 1.08) *p* = 0.08], SGA [OR: 1.57 (0.42, 5.80) *p* = 0.50], GA at delivery [MD: −0.09 week (−0.40, 0.21) *p* = 0.55], and preterm delivery [OR: 2.57 (0.95, 6.96) *p* = 0.06] (Figure S2). Also, no significant differences were noted in neonatal hypoglycemia [OR:0.85 (0.43, 1.68) *p* = 0.64], neonatal hyperbilirubinemia [OR: 0.99 (0.52, 1.89) *p* = 0.98], Apgar score at 5 min [OR-0.15 (−0.44, 0.14) *P* = 0.32], admission to the NICU [OR: 0.86 (0.49, 1.52) *p* = 0.60], and RDS [OR: 0.89 (0.25, 3.16) *p* = 0.85] (Figure S3). Although it was not statistically significant, the LGA showed a lower trend in the CGM group. Similarly, the need for insulin therapy was higher in the CGM group, but the difference was not significant. The funnel plot, presented in Supplementary Figure S4, did not indicate any significant publication bias. Egger’s test did not reveal significant publication bias across the outcomes assessed as shown in table S3.

### Sensitivity analysis and heterogeneity

The sensitivity analysis confirmed the robustness of the findings, as the effect estimates remained consistent with the primary meta-analysis across all outcomes. For maternal outcomes such as gestational age at delivery, caesarean section, and insulin therapy requirement, as well as neonatal outcomes including birth weight, macrosomia, hypoglycemia, hyperbilirubinemia, and NICU admission, the magnitude of the effect estimates did not materially change upon sensitivity analysis (Table S3, Figure S5 and S6).

Assessment of heterogeneity using the Cochrane Q test and I² statistics showed low heterogeneity for most outcomes (I² = 0%), indicating homogeneity across included studies. Exceptions were HbA1c (I² = 74%, τ² = 0.02), reflecting substantial heterogeneity (Table S3). This heterogeneity is likely attributable to differences in population characteristics (maternal age, BMI and glycemic status), use of varying diagnostic criteria for GDM across studies, the types of CGM devices employed, and inconsistencies in the duration and frequency of CGM use.

### Quality assessment

**Fig. 4:**
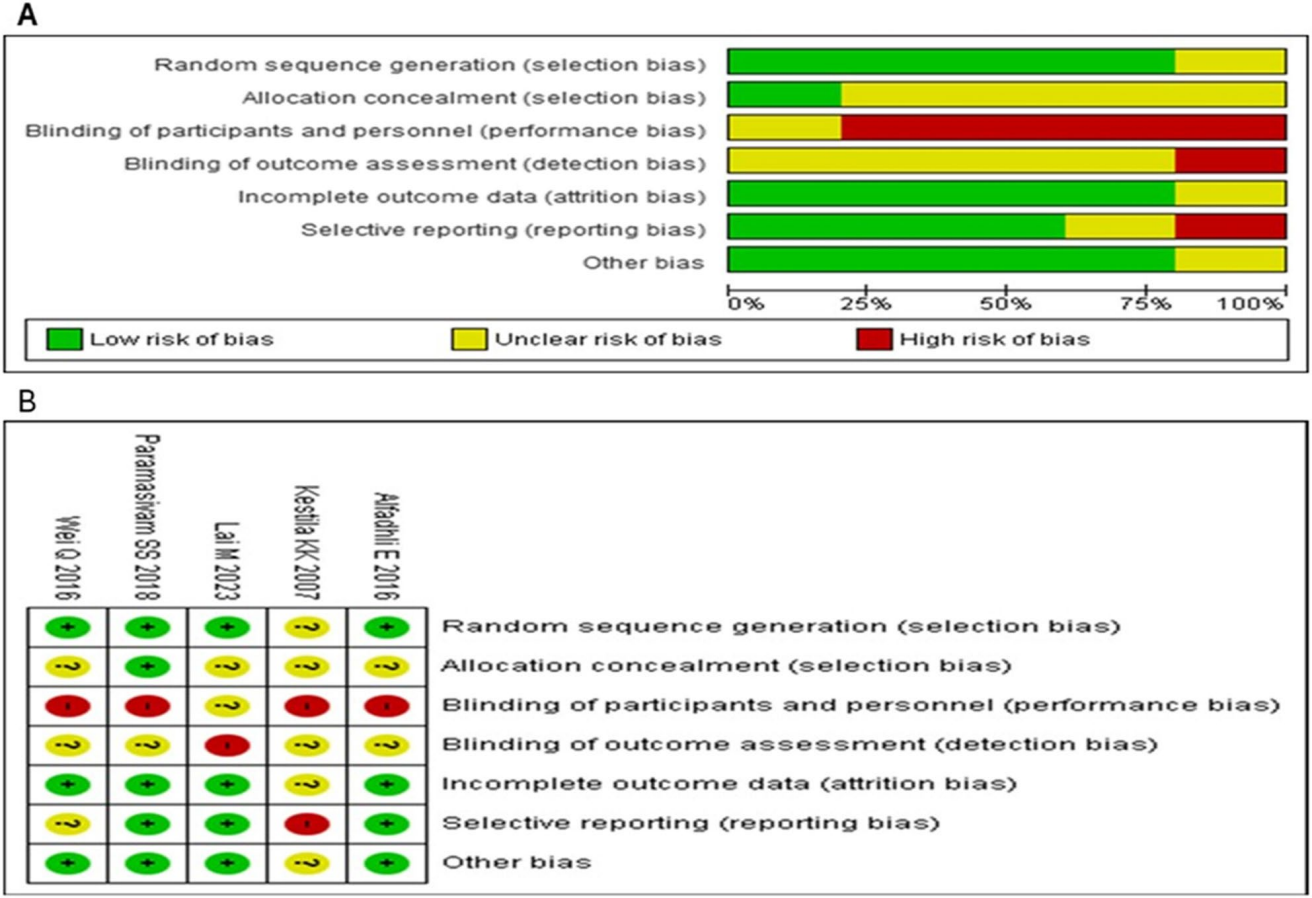
Risk of bias summary of included studies

The five trials included in the study were assessed to have a low risk of bias for random sequence generation, allocation of concealment, incomplete outcome data, and selective reporting. However, the blinding of participants and outcome assessment was rated as having a high risk of bias due to the nature of the intervention where blinding was difficult. The risk of bias graphs and summary are presented in Fig. 4.

## Discussion

This meta-analysis provides valuable insights into the comparative efficacy of CGM versus SMBG on maternal and neonatal outcomes in GDM. The meta-analysis showed reduction in HbA1c in GDM women on CGMS as compared to those on SMBG. The superior control also reflected in neonatal outcomes with the meta-analysis showing lower rates of macrosomia and lower birth weight in CGM group compared to SMBG. No significant differences were noted in the secondary maternal and neonatal outcomes between CGM and SMBG groups. Two studies [12, 13] that reported on time in range, time below range, and time above range indicated that these metrics remained within the recommended targets for pregnancy. Superior glycemic control with CGM could be explained by better understanding of glycemic patterns which could assist in intensifying the dietary modifications and pharmacotherapy in the GDM management. These findings contribute to understanding whether CGM offers significant benefits over SMBG, guiding healthcare providers in choosing the most appropriate monitoring strategy for optimal care of pregnant women with GDM.

Real-world data from large cohorts of women with GDM show that CGM use is linked to improved glycemic control, lower HbA1c, and favorable neonatal outcomes such as reduced rates of macrosomia and NICU admissions. These outcomes support findings from clinical trials and emphasize CGM’s practical utility in routine pregnancy care [14]. Several systematic reviews have examined CGM use during pregnancy, primarily including populations with T1DM and T2DM, which distinguishes them from our meta-analysis. Chang VY et al. reviewed studies involving GDM, T1DM, and T2DM populations, reporting a significant improvement in HbA1c levels in the CGM group [15]. In contrast, Wilkie G et al. focused exclusively on pregnant women with T2DM, evaluating LGA infants and pre-eclampsia as primary outcomes, with no significant differences between groups [16]. Similarly, Moy FM et al. included participants with T1DM and T2DM, assessing maternal glycaemic control (fasting blood glucose and HbA1c) and infant birthweight or macrosomia, but found no significant differences [17]. CGM was associated with significant reductions in HbA1c and birth weight, consistent with the findings of the previous meta-analysis. In contrast to that study, however, the present analysis also demonstrated a significant reduction in macrosomia, while secondary maternal and neonatal outcomes showed no significant differences, which is in line with earlier results [18]. A key distinction of this study is that it exclusively included primary studies focused solely on patients with GDM, excluding any studies where GDM was analyzed as a subgroup.

This meta-analysis is the first to specifically examine the use of CGM exclusively in women with GDM, thereby removing potential confounding effects from other types of diabetes. By isolating CGM’s impact on maternal and neonatal outcomes in GDM, our findings offer clearer, more actionable insights for healthcare providers, directly contributing to GDM-specific treatment guidelines and decision-making.

With the rising prevalence of GDM, effective tools are needed to reduce adverse maternal and fetal outcomes from poor glycemic control. SMBG has traditionally served as the standard method for glucose monitoring in patients with GDM [19]. However, adherence to prescribed testing regimens is often suboptimal due to the associated discomfort, inconvenience, and challenges in interpreting the data [20]. Moreover, SMBG frequently misses critical glycemic patterns, particularly premeal and nocturnal hypoglycemia, which can go undetected [21]. CGM offers a patient-centered approach by providing real-time feedback for diabetes management [22], though its use in GDM remains underexplored. CGM more effectively detects postprandial hyperglycemia than self-monitoring [11], captures glycemic excursions linked to fetal overgrowth [23], and identifies fluctuations such as nocturnal hypoglycemia often missed by intermittent monitoring. Time in range during later trimesters is strongly associated with LGA risk [12], underscoring CGM’s potential to guide timely treatment, reduce complications, and improve outcomes in GDM. Advances in CGM technology have improved accuracy, reduced calibration needs, and extended sensor wear, making it a reliable tool for pregnancy glucose monitoring [24]. Beyond tracking, CGM supports patient education, dietary planning, and behavioral feedback, with guidelines increasingly endorsing its use in cases of suboptimal control or hypoglycemia risk. Nonetheless, barriers such as cost, reimbursement issues, and the lack of pregnancy-specific standards limit wider adoption [25].

Clinicians managing pregnant patients with diabetes should develop expertise in interpreting CGM data, setting pregnancy-specific glycemic targets, and adjusting treatment accordingly to maximize the benefits of CGM during pregnancy. Recent studies emphasize the need for pregnancy-specific CGM metrics and the establishment of trimester-specific normative targets to enable earlier detection of dysglycemia, guide timely therapeutic interventions, and reduce adverse maternal-fetal outcomes [26, 27].

Our meta-analysis strengthens the existing evidence by refining the scope, introducing novel findings, and providing clinically relevant insights tailored specifically to the GDM population. The limitation of this meta-analysis is generalizability of the results which may be impacted by heterogeneity caused by differences in research design, population characteristics, diagnostic criteria, and intervention regimens among the trials.

## Conclusion

The study showed that women with GDM using CGM had reduced HbA1c levels, with lower rates of macrosomia and decreased birth weights, compared to those using SMBG. CGM presents a promising, patient-focused approach in improving glycemic control and minimizing adverse maternal and fetal outcomes. The future research should include larger, more diverse populations, establish normative glycemic metrics in pregnancy to improve the generalizability and reliability of findings, and confirm the clinical benefits of CGM. Additionally, evaluating CGM’s cost-effectiveness, enhancing device accessibility, and exploring patient adherence challenges will provide a more comprehensive understanding of its potential in managing GDM. Intensive glycemic control with use of technologies like CGM has the potential to transform diabetes management during pregnancy.

## Supplementary Information

Supplementary Material 1.

## Data Availability

Data is provided within the manuscript or supplementary information files.

## Abbreviations

CGM: Continuous Glucose Monitoring
GDM: gestational diabetes mellitus
SMBG: Self-Monitoring of Blood Glucose
RCTs: Randomized clinical trials
