# Supplementary material for: Effectiveness of continuous glucose monitoring on maternal and neonatal outcomes in gestational diabetes mellitus: a systematic review and meta-analysis
Source: BMC Pregnancy Childbirth. 2026 Jan 30;26:204. doi: 10.1186/s12884-026-08663-8 (PMC12934024; doi:10.1186/s12884-026-08663-8)
Supplement: Supplementary file 1 — Supplementary Material 1. [file 12884_2026_8663_MOESM1_ESM.docx]

**Table S1. Search strategy**

| **Database** | **Search query** | **Search result** |
| --- | --- | --- |
| Cochrane | "Continuous glucose monitoring" in Title Abstract Keyword OR CGM in Title Abstract Keyword AND "gestational diabetes mellitus" in Title Abstract Keyword OR GDM in Title Abstract Keyword | 650 |
| Scopus | ( TITLE-ABS-KEY ( continuous glucose monitoring ) OR TITLE-ABS-KEY ( cgm ) AND TITLE-ABS-KEY ( gestational diabetes mellitus ) OR TITLE-ABS-KEY ( gdm ) ) AND PUBYEAR > 1999 AND PUBYEAR < 2024 | 1204 |
| Embase | #1. (('continuous glucose monitoring'/exp OR 'continuous glucose monitoring' OR 'cgm') AND ('gestational diabetes mellitus'/exp OR 'gestational diabetes mellitus') OR 'gdm') AND  ('randomized controlled trial'/exp OR 'randomized controlled trial')  #2. #1 AND (2000:py OR 2001:py OR 2002:py OR 2003:py OR 2004:py OR 2005:py OR 2006:py OR 2007:py OR 2008:py OR  2009:py OR 2010:py OR 2011:py OR 2012:py OR 2013:py OR 2014:py OR 2015:py OR 2016:py OR 2017:py OR 2018:py OR 2019:py OR 2020:py OR 2021:py OR 2022:py OR 2023:py OR 2024:py)  #3. #2 AND 'Article'/it | 849 |
| PubMed | (((("continuous glucose monitoring"[MeSH Terms] OR ("continuous"[All Fields] AND "glucose"[All Fields] AND "monitoring"[All Fields]) OR "continuous glucose monitoring"[All Fields] OR "CGM"[All Fields]) AND ("diabetes, gestational"[MeSH Terms] OR ("diabetes"[All Fields] AND "gestational"[All Fields]) OR "gestational diabetes"[All Fields] OR ("gestational"[All Fields] AND "diabetes"[All Fields] AND "mellitus"[All Fields]) OR "gestational diabetes mellitus"[All Fields])) OR "GDM"[All Fields]) AND ("randomized controlled trial"[Publication Type] OR "randomized controlled trials as topic"[MeSH Terms] OR "randomized controlled trial"[All Fields] OR "randomised controlled trial"[All Fields])) AND ((clinicaltrial[Filter] OR randomizedcontrolledtrial[Filter] OR systematicreview[Filter]) AND (2000:2024[pdat])) | 672 |

**Table S2. Summary table of diagnostic criteria used in included studies**

| **Study** | **Diagnostic criteria / Glucose Load** | **Gestational weeks**  **for OGTT** | **Diagnosis Rule** | **Thresholds** | | |
| --- | --- | --- | --- | --- | --- | --- |
|  |  |  |  | **FBG** | **1-hour PPBS** | **2-hour PPBS** |
| Alfadhli et al., 2016 | IADPSG criteria – 75 g OGTT | 22 - 34 | At least two abnormal high plasma glucose values out of three measurements. | ≥ 5.1 mmol/L | ≥ 10.0 mmol/L | ≥ 8.5 mmol/L |
| Kestilä et al., 2007 | Criteria applied to a high-risk group according to the evaluation system used in Finland - 75 g OGTT | 22 - 34 | At least two abnormal high plasma glucose values out of three measurements. | > 5.1 mmol/l. | > 10.0 mmol/l | > 8.7 mmol/l. |
| Lai et al., 2023 | IADPSG criteria - 75 g OGTT | 24 - 28 | If one or more plasma glucose values out of three measurements. | ≥ 5.1 mmol/L | ≥ 10.0 mmol/L | ≥ 8.5 mmol/L |
| Paramasivam et al., 2018 | Local guidelines -75 g OGTT | - | If OGTT reveals a fasting plasma glucose or a 2-h post-load plasma glucose higher than the threshold. This study specifically enrolled women with confirmed GDM using these criteria. | ≥ 5.1 mmol/L | - | ≥ 7.8 mmol/L |
| Wei et al., 2016 | ADA criteria - 75 g OGTT | 24 - 28 | At least one abnormally high plasma glucose value out of the three measurements. | > 92 mg/dL (5.1 mmol/L) | > 180 mg/dL (10.0 mmol/L) | > 153 mg/dL (8.5 mmol/L). |

Note: OGTT - Oral Glucose Tolerance Test, ADA - American Diabetes Association, IADPSG - International Association of the Diabetes and Pregnancy Study Groups.

| 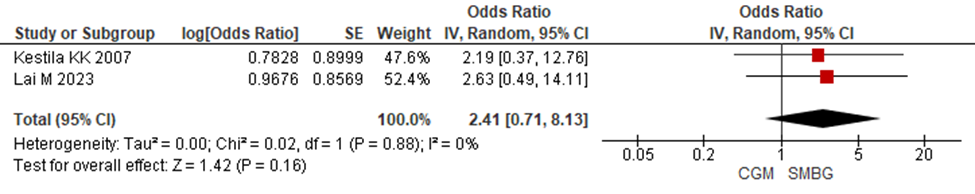 |
| --- |
| 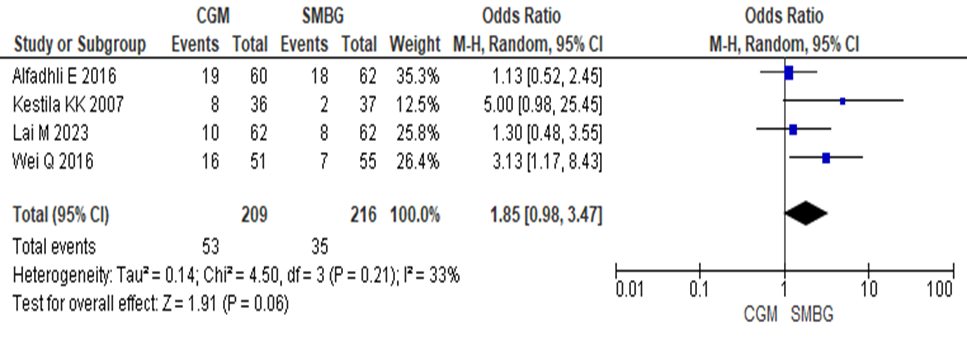 |
| 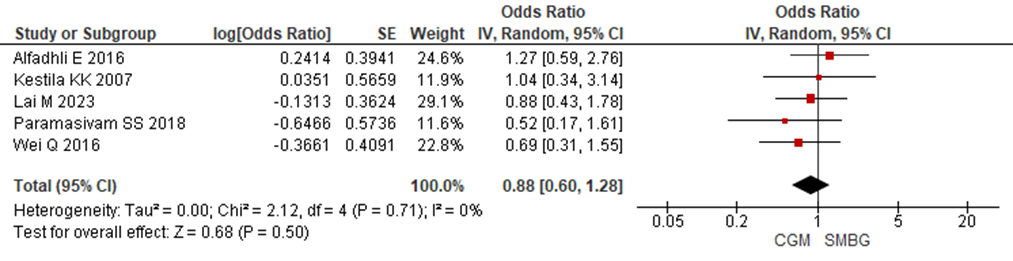 |

**Figure S1.** **Forest Plot for maternal outcomes (A. Gestational hypertension, B. patient requiring insulin therapy and C. Caesarean section) between the CGM and SMBG Groups.**

| 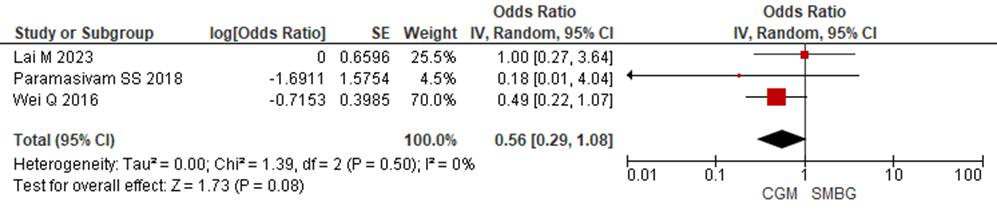 |
| --- |
| 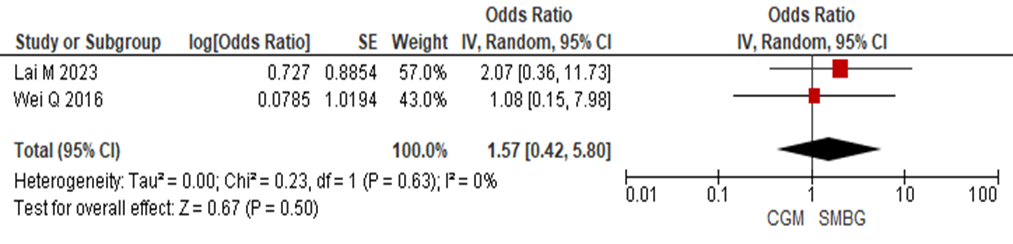 |
| 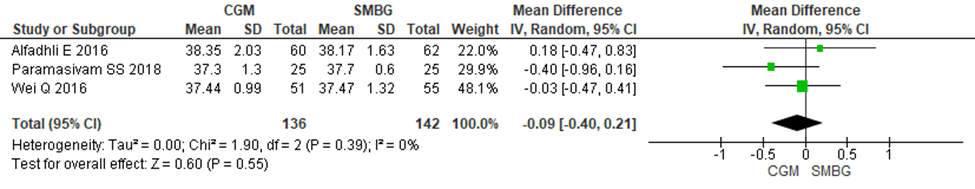 |
| 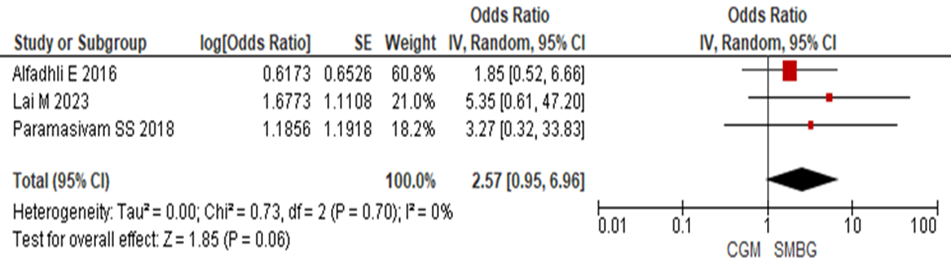 |

**Figure S2.** **Forest Plot for neonatal outcomes (A. LGA, B. SGA, C. GA at delivery and D. Preterm delivery) between the CGM and SMBG Groups.**

| 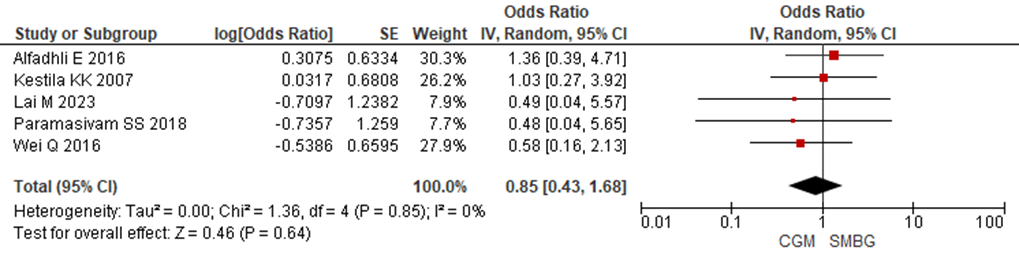 |
| --- |
| 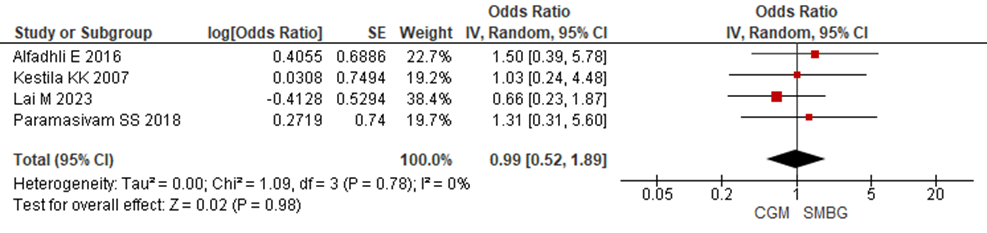 |
| 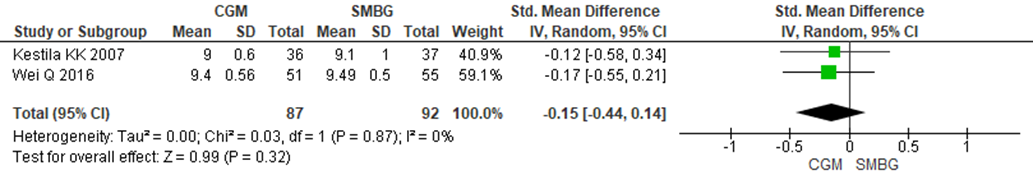 |
| 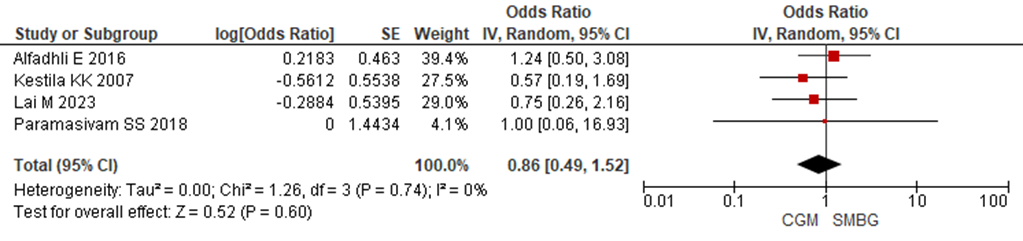 |
| 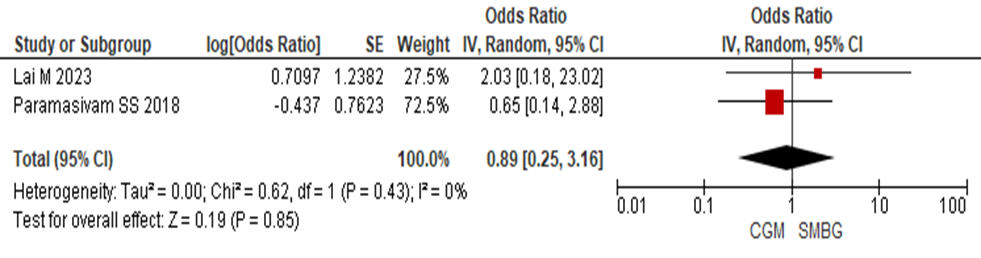 |

**Figure S3.** **Forest Plot for neonatal outcomes (A. neonatal hypoglycemia, B. neonatal hyperbilirubinemia, C. Apgar score, D. NICU admission and E. RDS) between the CGM and SMBG Groups.**


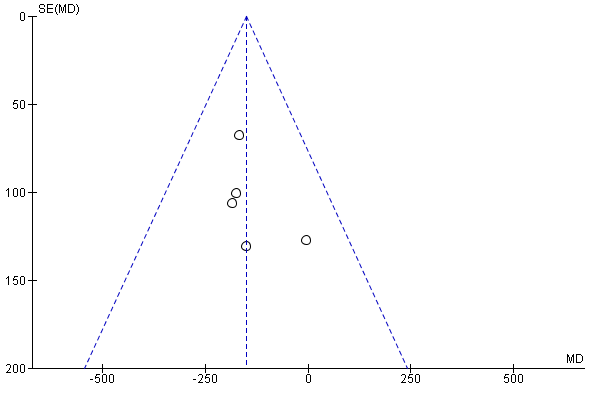


**Figure S4.** **Funnel plot of included studies.**

| **HbA1c**  **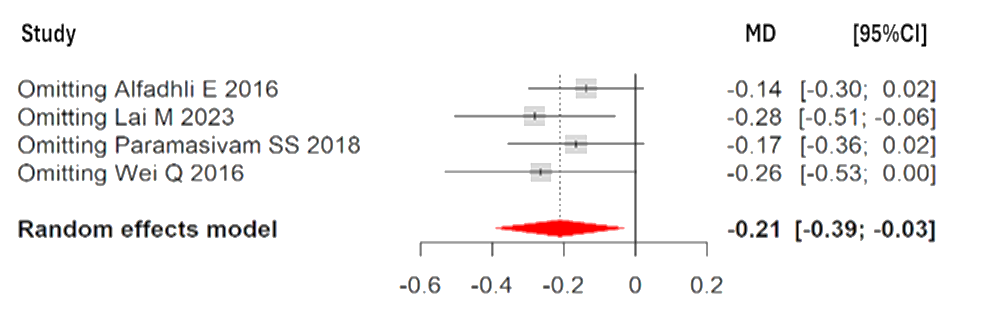** |
| --- |
| **Macrosomia**  **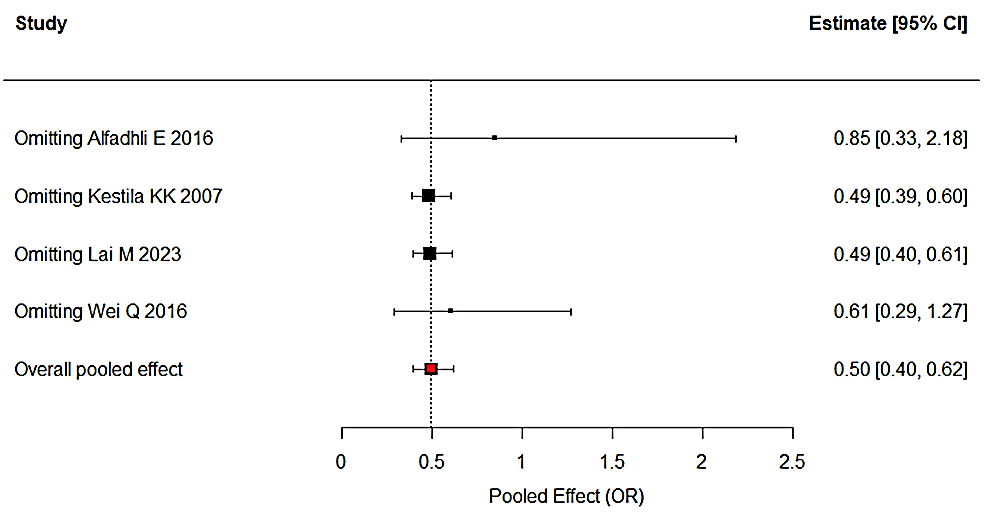** |
| **Birth weight**  **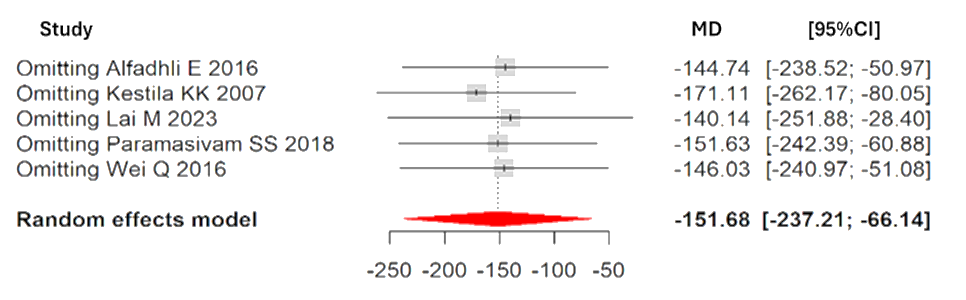** |
| **Patient requiring insulin therapy**  **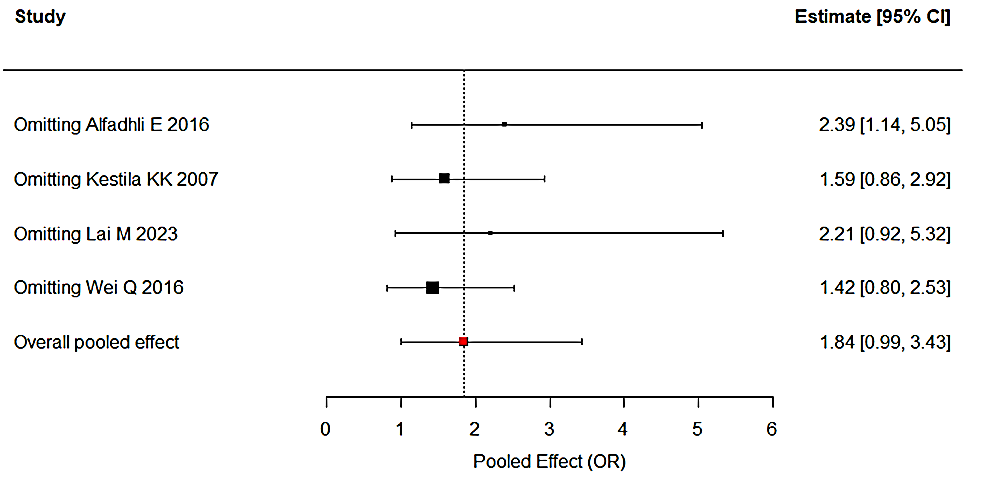** |
| **Caesarean section**  **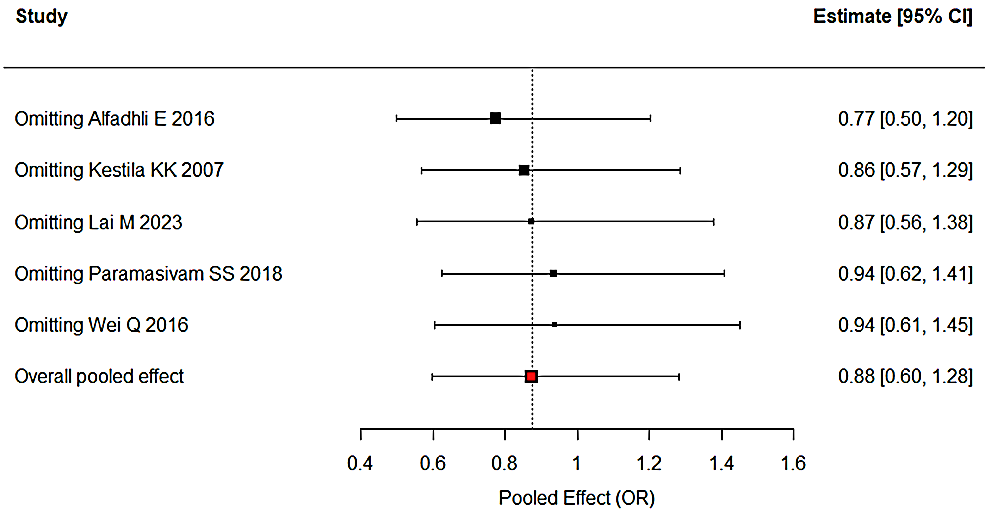** |
| **GA at delivery**  **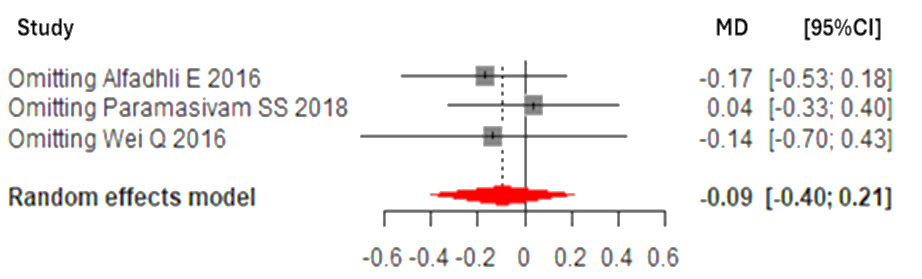** |

**Figure S5. Sensitivity analysis forest plot.** Note**: GA -** Gestational Age, **HbA1c** - Glycated Hemoglobin, OR - Odds Ratio

| **Large for gestational age**  **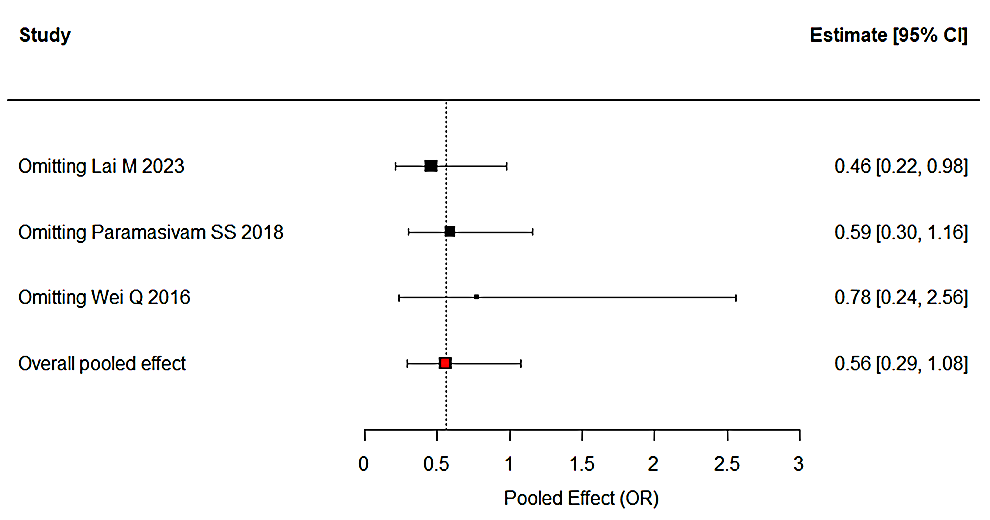** |
| --- |
| **Preterm delivery**  **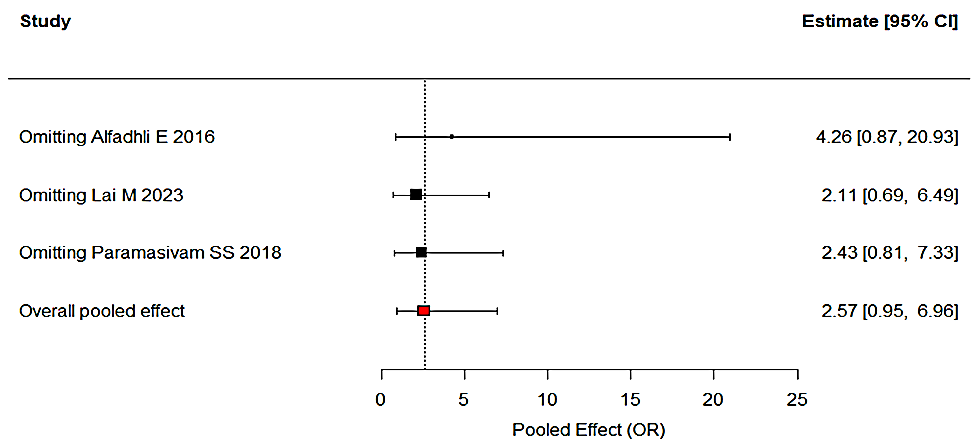** |
| **Neonatal hypoglycemia**  **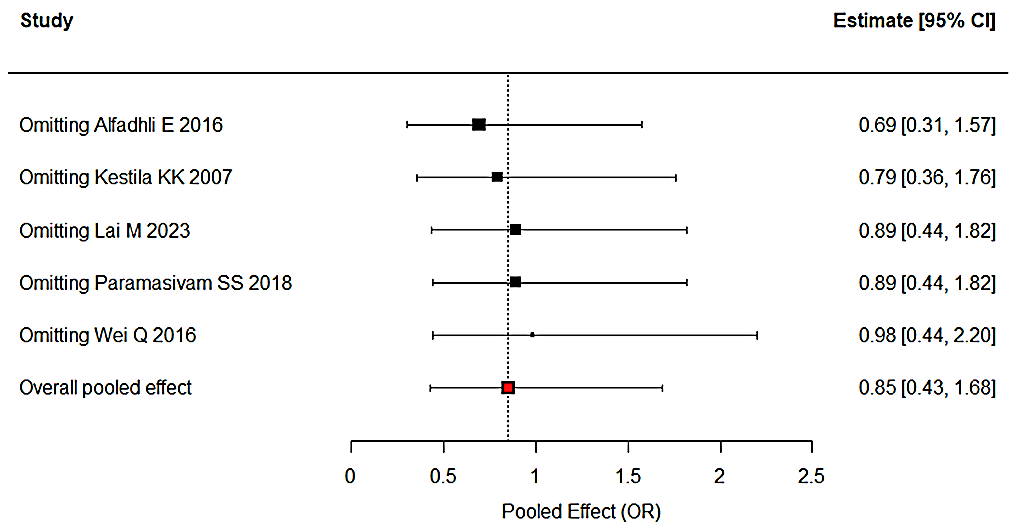** |
| **Neonatal hyperbilirubinemia**  **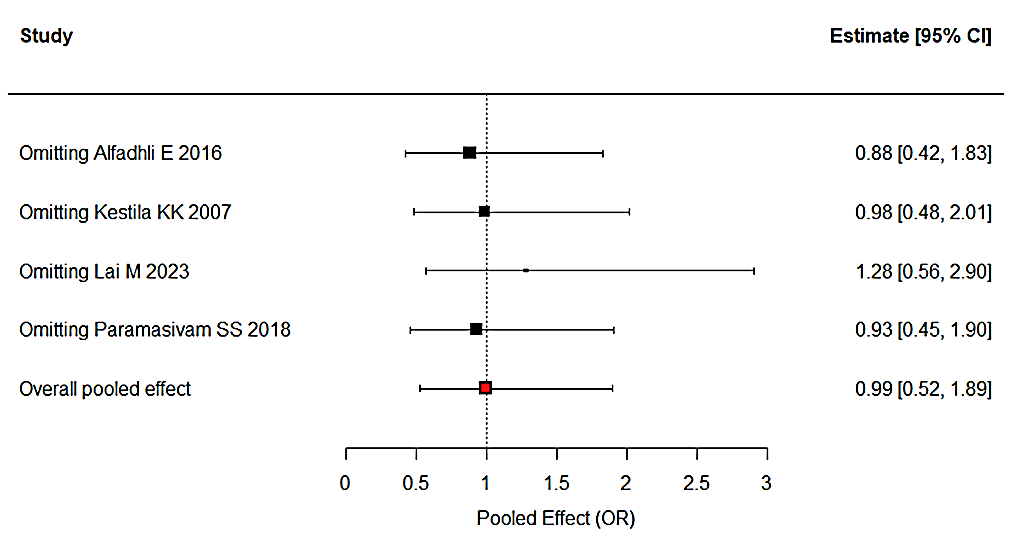** |
| **NICU admission**  **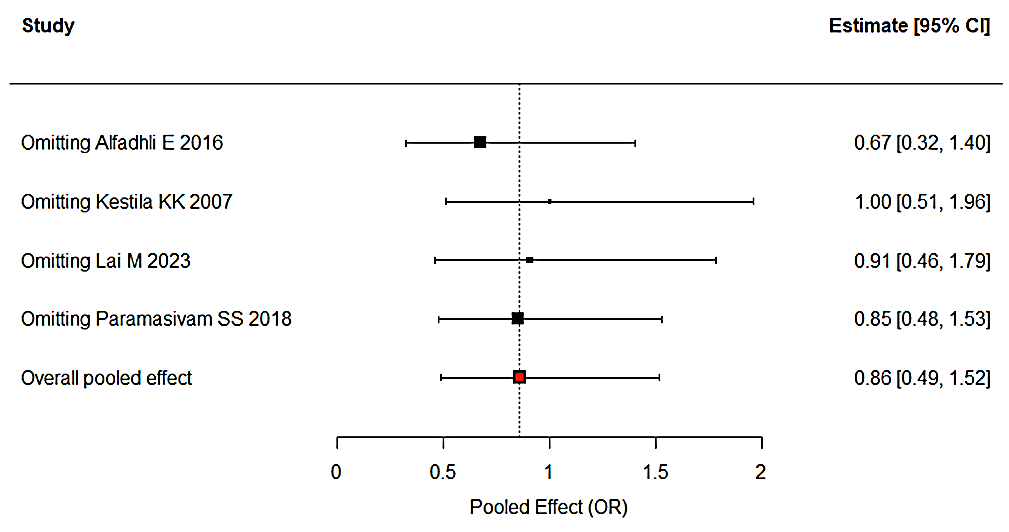** |

**Figure S6. Sensitivity analysis forest plot.** Note**: NICU** - Neonatal Intensive Care Unit, OR -Odds Ratio

**Table S3. Summary of sensitivity analyses and heterogeneity across all outcomes**

| **Outcomes** | **Studies** | **Sensitivity analysis** | | | **tau2** | **I^2^(%)** | **Cochrane Q test** | | **Egger’s test** |
| --- | --- | --- | --- | --- | --- | --- | --- | --- | --- |
|  |  | **Effect estimate** | **95%CI (low)** | **95%CI (High)** |  |  | **Chi^2^** | **p value** | **P value** |
| HbA1c | 4 | -0.21 | -0.39 | -0.03 | 0.02 | 74 | 11.54 | 0.01 | 0.12 |
| Birth weight | 5 | -151.70 | -237.20 | -66.14 | 0 | 0 | 1.53 | 0.82 | 0.41 |
| Macrosomia | 4 | 0.50 | 0.40 | 0.62 | 0 | 0 | 2.08 | 0.56 | 0.23 |
| GA at delivery | 3 | -0.09 | -0.40 | 0.21 | 0 | 0 | 1.90 | 0.39 | 0.93 |
| Preterm delivery | 3 | 2.57 | 0.95 | 6.96 | 0 | 0 | 0.73 | 0.70 | 0.45 |
| Caesarean section | 5 | 0.88 | 0.60 | 1.28 | 0 | 0 | 2.12 | 0.71 | 0.60 |
| Neonatal hypoglycemia | 5 | 0.85 | 0.43 | 1.68 | 0 | 0 | 1.36 | 0.85 | 0.47 |
| Neonatal Hyperbilirubinemia | 4 | 0.99 | 0.52 | 1.89 | 0 | 0 | 1.09 | 0.78 | 0.38 |
| LGA | 3 | 0.56 | 0.29 | 1.08 | 0 | 0 | 1.39 | 0.50 | 0.72 |
| NICU | 4 | 0.86 | 0.49 | 1.52 | 0 | 0 | 1.26 | 0.74 | 0.89 |
| Patient requiring Insulin therapy | 4 | 1.84 | 0.99 | 3.43 | 0.14 | 33 | 4.49 | 0.21 | 0.10 |

Note**:** CI – Confidence interval, **GA –** Gestational Age, **HbA1c** - Glycated Hemoglobin, **LGA** - Large for Gestational Age, **NICU** - Neonatal Intensive Care Unit.
